# Inhibitors of nuclear factor kappa B cause apoptosis in cultured macrophages

**DOI:** 10.1080/09629359791721

**Published:** 1997-06

**Authors:** E. E. Mannick, J. Mishra, J. Marque, M. Clavell, M. J. S. Miller, P. D. Oliver

**Affiliations:** 1 Department of Pediatrics Louisiana State University New Orleans LA 70112 USA

## Abstract

The precise role of the transcription factor nuclear factor kappa B (NF- κB) in the regulation of cell survival and cell death is still unresolved and may depend on cell type and position in the cell cycle. The aim of this study was to determine if three pharmacologic inhibitors of NF-κB, pyrrolidine dithiocarbamate, N-tosyl-L-lysl chloromethyl ketone and calpain I inhibitor, induce apoptosis in a murine macrophage cell line (RAW 264.7) at doses similar to those required for NF-κB inhibition. We found that each of the three inhibitors resulted in a dose- and time-dependent increase in morphologic indices of apoptosis in unstimulated, LPS-stimulated and TNF-stimulated cells. Lethal doses were consistent with those required for NF- κB inhibition. We conclude that nuclear NF-κB activation may represent an important survival mechanism in macrophages.


Mediators of Inflammation, 6, 225–232 (1997)

### Inhibitors of nuclear factor kappa B cause apoptosis in cultured macrophages

E. E. Mannick,[CA] J. Mishra, J. Marque, M. Clavell, M. J. S. Miller, P. D. Oliver

Department of Pediatrics, Louisiana State University, New Orleans, LA 70112, USA

[CA]Corresponding Author
Tel: (+1) 504 568 8366
Fax: (+1) 504 568 7532

**The precise role of the transcription factor nuclear factor kappa B (NF-κB) in the regulation of cell survival and cell death is still unresolved and may depend on cell type and position in the cell cycle. The aim of this study was to determine if three pharmacologic inhibitors of NF-κB, pyrrolidine dithiocarbamate, N-tosyl-L-lysl chloromethyl ketone and calpain I inhibitor, induce apoptosis in a murine macrophage cell line (RAW 264.7) at doses similar to those required for NF-κB inhibition. We found that each of the three inhibitors resulted in a dose- and time-dependent increase in morphologic indices of apoptosis in unstimulated, LPS-stimulated and TNF-stimulated cells. Lethal doses were consistent with those required for NF-κB inhibition. We conclude that nuclear NF-κB activation may represent an important survival mechanism in macrophages.**

**Key words:** Apoptosis, Macrophage, Nuclear factor κB

### Introduction

Nuclear factor kappa B (NF-κB) is a complex of dimeric transcription factors (p50, p65, c-rel, Rel B) activated by tumor necrosis factor (TNF), reactive oxygen species, phorbol myristate acetate (PMA), lipopolysaccharide (LPS), as well as serine/threonine and tyrosine kinases. NF-κB plays a significant role in immunity and inflammation; genes regulated by NF-κB include interleukins 1, 2, 6 and 8, inducible nitric oxide synthase, the major histocompatibility complex Class I, the adhesion molecules, ICAM, VCAM and ELAM, and the immunoglobulin κ light chain (for a review see reference 1). In addition, there is now data to suggest that NF-κB may also play a role in the regulation of cell survival, cell death and oncogenesis:[2] for example, inhibition of p65, a transcriptionally active subunit of NF-κB, with antisense messenger RNA has been shown to inhibit tumorigenicity of transformed cell lines and tumor growth in animals;[3] Bcl-3, a molecule related to I-κB and which is unable to inhibit nuclear binding of the p65 subunit, has been linked to the development of B-cell lymphomas.[4]

The precise role of NF-κB in the regulation of cell death is still a matter of controversy. Initially, several lines of evidence appeared to indicate a role for the excessive activation of NF-κB as a cause of apoptosis: nuclear binding of transcriptionally active NF-κB p50/p65 heterodimers was noted to follow initiation of apoptosis in a variety of cell lines,[5–8] and three distinct pharmacologic inhibitors of NF-κB, pyrrolidine dithiocarbamate (PDTC), *N*-tosyl-L-lysyl chloromethylketone (TPCK) and *N*-acetyl cysteine, were shown to inhibit apoptosis in HL-60 cells, human thymocytes, prostate carcinoma and neuroblastoma cell lines.[9,10] In some of these experiments, however, the doses of these agents required to inhibit apoptosis were lower than those required to inhibit NF-κB, suggesting that their protective effects were not necessarily due to their ability to inhibit NF-κB.[11] The ability of the TNF-receptor both to activate NF-κB and to execute an apoptosis program were initially suspected to be causally related, futher supporting a pro-apoptotic function for NF-κB. Recent studies, however, have demonstrated that the initiation of apoptosis and the activation of NF-κB by TNF are separate, and possibly, mutually exclusive events.[12–14]

In spite of the fact that various compounds which share the ability to inhibit NF-κB including PDTC, *N*-acetyl cysteine, genistein, dexamethasone and staurosporine can cause apoptosis at doses similar to those required for NF-κB inhibition,[15–21] inhibition of NF-κB itself had not been proposed as a mechanism of apoptosis until very recently. The first evidence in favor of a pro-survival role for NF-κB came from a study of mice with a homozygous deletion of the gene for the p65 component of NF-κB who underwent early embryonic death characterized by apoptosis of liver cells.[22] A second piece of evidence came from the finding of an NF-κB binding site on the promoter region

© 1997 Rapid Science Publishers of A20, a zinc finger protein which inhibits apoptosis.[23] Finally, three separate reports were published recently which demonstrated prevention of TNF-mediated apoptosis by induction of NF-κB *in vitro*.[24–26] In a fourth study, the NF-κB inhibitor, TPCK was found to induce apoptosis in unstimulated WEHI B-cell lymphoma cells.[27]

Although this evidence supports a pro-survival role for NF-κB, other recent studies have demonstrated that the precise relationship between NF-κB and cell death is still unresolved. Grilli *et al.* for example, have reported that aspirin and sodium salicylate protect against glutamate-induced apoptosis by inhibiting NF-κB in preparations of primary neurons.[28] Similarly, Tsai *et al.* have shown that PDTC protects neurons from cell death at the same doses at which it causes apoptosis in smooth muscle cells.[15] These conflicting results point to the need for further studies to define the conditions and cell types in which NF-κB inhibitors either cause or inhibit apoptosis. The answers to these questions are of particular importance because NF-κB inhibitors have been proposed as potential therapeutic agents for inflammatory disease[29] and cancer.[25]

The aim of the current study was to determine if three pharmacologically distinct inhibitors of NF-κB (PDTC, TPCK, and Calpain I inhibitor) induce apoptosis in a transformed murine macrophage cell line at doses required for the inhibition of NF-κB. We also sought to determine if activation of the cells with either TNF, LPS or PMA could reverse the effects of NF-κB inhibitors.

### Materials and Methods

### Reagents

RAW 264.7 cells, from a transformed murine peritoneal macrophage cell line, were obtained from ATCC (Rockville, MD); recombinant murine TNF-α was purchased from R&D Systems (Minneapolis, MN); [$\gamma$-$^{32}$P]ATP and Hyperfilm were obtained from Amersham Life Sciences (Arlington Heights, IL); calpain I inhibitor was purchased from ICN (Costa Mesa, CA); Cellular DNA Flow Cytometric Analysis Kit was obtained from Boehringer Mannheim (Indianapolis, IN); polynucleotide kinase and kinase buffer for electrophoretic mobility shift assays were obtained from Promega (Madison, WI); NF-κB consensus oligonucleotide was obtained from Stratagene (La Jolla, CA); fetal calf serum was purchased from HyClone (Logan, UT). All other reagents were purchased from Sigma (St. Louis, MO).

### Cell culture

RAW 264.7 cells were cultured in Dulbecco's modified Eagle's medium with 10% fetal calf serum containing < 0.6 E.U. lipopolysaccharide (LPS), penicillin (100 units/ml) streptomycin (100 μg/ml) and $NaHCO_3$ (2·2 g/l). Cells were exposed for 18 h to varying doses of each inhibitor as follows: PDTC, 0, 25, 50, 100 μM; TPCK, 0, 10, 25, 50 μM; and calpain I inhibitor, 0, 5, 10, 25 μM. In addiitonal experiments, cells were exposed for 6, 12, 18 or 24 h to either 100 μM PDTC, 50 μM TPCK, 10 μM calpain I inhibitor or vehicle (control). Some cells were concomitantly exposed to 10 nM phorbol myristate acetate (PMA), 3 ng/ml TNF, 1 μg/ml LPS or 10 μg/ml of cycloheximide (CHX). Cells were then rinsed in phosphate buffered saline (PBS) or Tris buffered saline (TBS), scraped and pelleted in preparation for cell death analysis or nuclear extracts, respectively.

### Electrophoretic mobility shift assay

Nuclear extracts were prepared as previously described.[30] After determining the protein content of nuclear assays using a standard assay (Bio-RAD, Hercules, CA), 2 μg of each sample was incubated for 15 min with 5 μl of binding buffer (5 × TBE; 25% glycerol), 1 μl poly dI:dC (0·25 μg/μl) and 1 μl 50 mM dithiothreitol. Samples were then incubated for 30 min with a $^{32}$P-labeled consensus NF-κB binding oligonucleotide. To ensure specificity of binding, negative control samples were coincubated with unlabeled oligonucleotide ('cold competitor'). One μl of gel 10 × loading buffer (250 mM Tris-HCl, pH 7.5, 0.2% bromophenol blue, 40% glycerol) was added to each sample and samples were loaded on a 9% polyacrylamide nondenaturing gel and run at 190 V for 3–4 h in 0.25 × TBE. Gels were exposed to X-ray film at −70°C overnight.

### Cell death assays

Cell death by apoptosis was quantified by one of two methods. Cells from all treatment groups were resuspended in PBS containing 5 μg/ml of acridine orange and examined under a fluorescent microscope for morphologic changes consistent with apoptosis (nuclear condensation and fragmentation, membrane blebbing). The presence of apoptosis was confirmed by performing the TUNEL end-labeling assay which measures double-stranded DNA breaks by flow cytometry.[31] Briefly, cells were washed twice in cold PBS/1% BSA and then fixed with 100 μl of

4% paraformaldehyde sollution for 30 min. Cells were centrifuged at 188 × *g*, washed twice with PBS/1% BSA, and resuspended in 100 μl of permeabilization solution (0.1% Triton X-100 in 0.1% sodium citrate) for 2 min on ice. After washing twice, cells were resuspended in 50 μl of TUNEL reaction mixture (terminal deoxynucleotidyl transferase and nucleotide mixture) and incubated for 60 min at 37°C in a dark, humidified chamber. After washing samples twice in PBS, cells were resuspended in 250–500 μl of PBS and analyzed by flow cytometry. Cells undergoing apoptosis were expressed as a percentage of total cells.

### Statistical analysis

Comparisons among treatment groups were made by ANOVA and the Tukey–Kramer Multiple Comparisons Test using Instat (GreaftPad Software, San Diego, CA) software.

### Results

### Induction of apoptosis by NF-κB inhibitors

Apoptosis was assessed at 18 h for each inhibitor. Treatment with each of the three inhibitors resulted in an increase in the percentage of cells undergoing apoptosis as assessed by acridine orange (Fig. 1) and TUNEL staining (Fig. 2). Compared to no treatment, PDTC, TPCK, and calpain I inhibitor each caused significant, dose-dependent increases in apoptosis as assessed by acridine orange staining (Fig. 1). Calpain I inhibitor was the most effective of the three agents at killing cells with an $EC_{50}$ of between 5 and 10 μM. PDTC was the least effective of the three agents with an $EC_{50}$ of approximately 100 μM. The $EC_{50}$ for TPCK was approximately 40 μM (Fig. 1). Apoptosis induction by each of the inhibitors at 18 h was confirmed using flow cytometry and the TUNEL assay (Fig. 2).

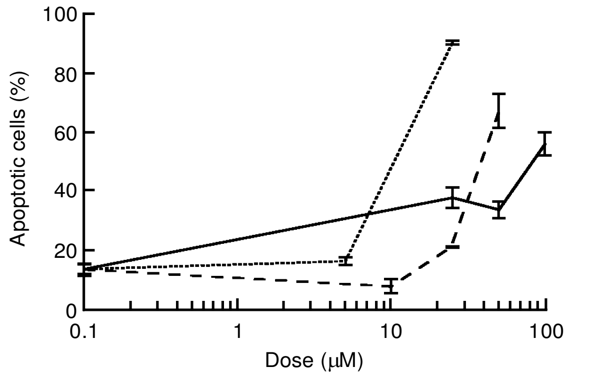

FIG. 1. Dose-response curves for apoptosis caused by NF-κB inhibitors. RAW 264.7 cells were incubated for 18 h with doses of PDTC (——), TPCK (- - - - -) and calpain I inhibitor (. . . . .) in the range 0–100 μM. Dosage data is plotted on a log scale. Percentage of apoptotic cells was quantified by counting the number of cells with morphologic characteristics of apoptosis per 100 cells using acridine orange staining. Calpain I inhibitor (25 μM), TPCK (50 μM) and PDTC (100 μM) all caused highly significant ($P < 0.001$) increases in apoptosis compared to controls. Error bars represent SEM. Each experiment was repeated at least three times.

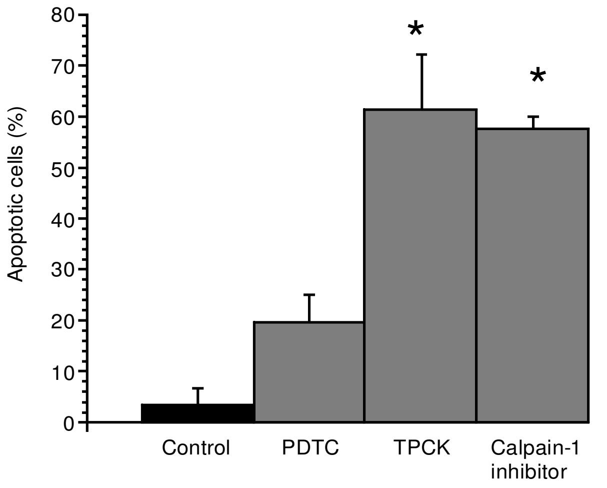

FIG. 2. Effects of NF-κB inhibitors on apoptosis measured by TUNEL/flow cytometry. RAW 264.7 cells were incubated for 18 h with either no treatment, 100 μM PDTC, 50 μM TPCK, or 10 μM calpain I inhibitor. The percentage of cells undergoing apoptosis was quantified by flow cytometry using TUNEL staining and is depicted on the ordinate. *, significant increases in apoptosis compared to controls ($P < 0.05$). Error bars represents SEM. Each experiment was repeated at least three times.

### Time course of apoptosis

The mean percentage of cells undergoing apoptosis as assessed by acridine orange staining at 6, 12, 18 and 24 h for each inhibitor was calculated (Fig. 3). Maximal induction of apoptosis by TPCK (50 μM) and PDTC (100 μM) occurred at 18 h. Calpain I inhibitor (10 μM) induced maximal killing at 12 h. Apoptosis became detectable at about 6 h post-treatment and began to decrease by 24 h post-treatment for all inhibitors. There was little morphologic evidence of apoptosis prior to 6 h for any of the inhibitors tested (data not shown).

### Effect of PMA

Experiments were conducted to determine if induction of apoptosis by NF-κB inhibitors could be overcome by concurrent signals which normally induce NF-κB. Neither TNF nor LPS afforded significant protection against cell death caused by any of the three inhibitors (data not shown). PMA (10 nM) led to a reduction in the amount of apoptosis induced by PDTC

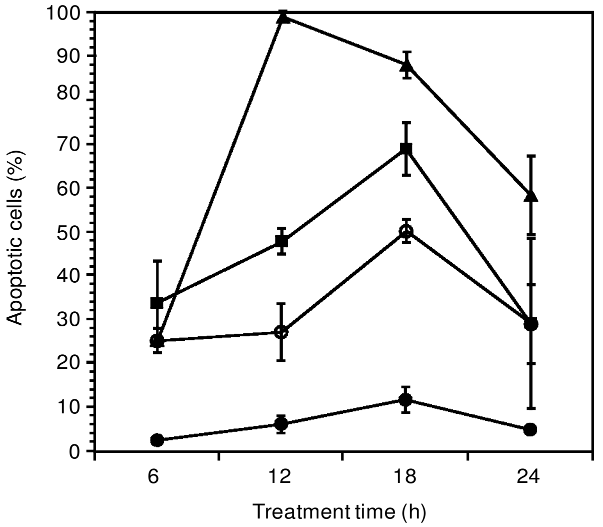

FIG. 3. Apoptosis caused by NF-κB inhibitors as a function of time. RAW 264.7 cells were incubated for 6, 12, 18 and 24 h with no treatment (●), 100 μM PDTC (○), 50 μM TPCK (■) or 10 μM calpain 1 inhibitor (▲). The percentage of cells undergoing apoptosis was assessed by acridine orange staining and is shown on the ordinate. Error bars represent SEM. Each experiment was repeated at least three times.

(100 μM), TPCK (50 μM) and calpain I inhibitor (10 μM) (Fig. 4). Only in the case of calpain I inhibitor, however, was this reduction significant ($P < 0{\cdot}001$).

### Effect of cycloheximidine

Cycloheximide has been reported to both prevent and induce apoptosis, presumably depending on whether it inhibits transcription of death genes or of survival genes.[32,33] In our experiments, cycloheximidine (10 μg/ml) offered no protection against apoptosis caused by PDTC (100 μM), TPCK (50 μM) and calpain I inhibitor (10 μM) at 18 h (Fig. 5). By itself, however, cycloheximide also induced extensive apoptosis at 18 h. These results suggest that cycloheximide may be blocking induction of a pro-survival gene in an additive fashion with NF-κB inhibitors.

### Effects of inhibitors on NF-κB binding by EMSA

To determine if apoptosis occurred at the same doses as NF-κB inhibition by PDTC, TPCK and calpain I inhibitor, electrophoretic mobility shift assays were performed using nuclear extracts from cells incubated for 18 h with different doses of each of the three inhibitors (Fig. 6). PDTC, at doses of 50 μM and higher, effectively inhibited the upper band of the two-band NF-κB characteristically seen on EMSA gels. The upper band corresponds to p50–p65 heterodimers. The lower band, which was not inhibited by PDTC, corresponds to p50–p50 homodimers, considered to be transcriptionally inactive. At doses above 10 μM, TPCK inhibited the bands corresponding to both the p50–p65 heterodimer and the p50–p50 homodimer. Interestingly, in some experiments, there was formation of a new band of higher molecular weight than the p50–p65 heterodimer which appeared at doses

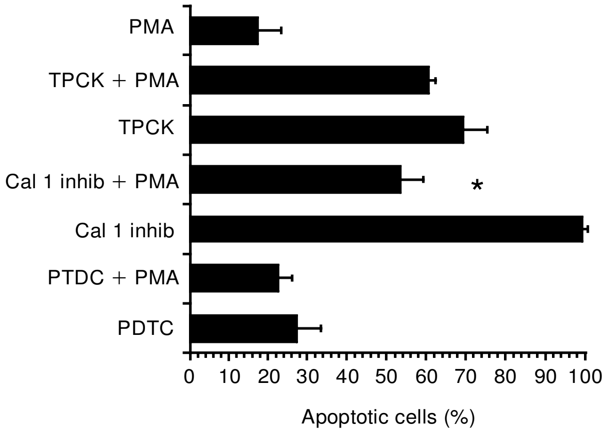

FIG. 4. Effects of PMA on apoptosis induced by NF-κB inhibitors. RAW 264.7 cells were incubated for 18 h with either PMA (10 nM), PDTC (100 μM), TPCK (50 μM), calpain I inhibitor (10 μM) or PMA with PDTC, TPCK with PMA or calpain I inhibitor with PMA. The mean percentage of cells undergoing apoptosis as assessed by acridine orange staining is represented on the abscissa. Error bars represent SEM. *, significant reduction in apoptosis in cells treated with a combination of calpain I inhibitor and PMA compared to cells treated with calpain I inhibitor alone ($P < 0{\cdot}05$). Each experiment was repeated at least three times.

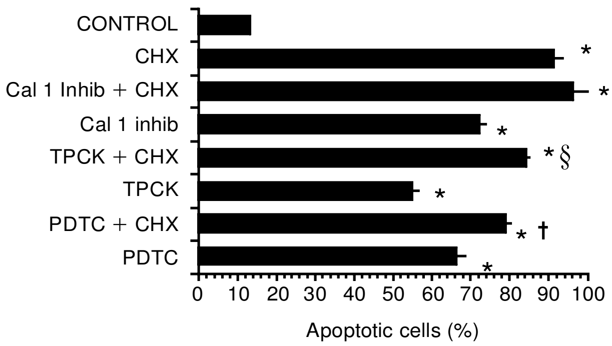

FIG. 5. Effects of cycloheximide (CHX) on apoptosis induced by NF-κB inhibitors. RAW 264.7 cells were incubated for 18 h with CHX (10 μg/ml), calpain I inhibitor (10 μM), TPCK (25 μM) or PDTC (100 μM). The percentage of cells undergoing apoptosis as assessed by acridine orange staining is shown on the abscissa. Error bars represent SEM. *, significant increase in apoptosis ($P < 0{\cdot}001$) in treated cells compared to control cells; §, significant increase ($P < 0{\cdot}001$) in apoptosis in cells treated with TPCK and CHX compared to cells treated with TPCK alone; + significant increase ($P < 0{\cdot}01$) in apoptosis in cells treated with both PDTC and CHX compared to cells treated with PDTC alone. Each experiment was repeated at least three times.

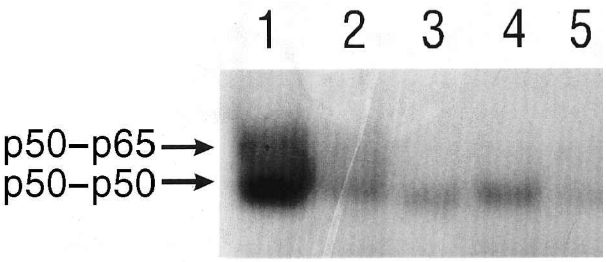

FIG. 6. Electrophoretic mobility shift assay – Effects of NF-κB inhibitors on NF-κB binding. RAW 264.7 cells were incubated for 2 h with either no treatment (lane 1); PDTC, 25 μM (lane 2); PDTC, 50 μM (lane 3); PDTC, 100 μM (lane 4); TPCK, 5 μM (lane 6); TPCK, 10 μM (lane 7); TPCK, 25μM (lane 8); TPCK, 50 μM (lane 9); calpain I inhibitor, 1 μM (lane 10); calpain I inhibitor, 5 μM (lane 11); calpain I inhibitor, 10 μM (lane 12); calpain I inhibitor, 25 μM (lane 13). Bands corresponding to p50–p65 heterodimers and to p50–p50 homodimers are indicated by arrows. The uppermost band seen in lanes 7, 9 and 12 is unidentified. This experiment was repeated at least three times.

of TPCK of 10 μM and higher. Calpain I inhibitor inhibited NF-κB (both p50–p50 and p50–p65 dimers) binding at doses of 1 μM and higher. As with TPCK, an unidentified higher molecular weight band appeared at doses of 10 μM calpain I inhibitor and higher.

These results show that the $EC_{50}$ for the induction of apoptosis by all three inhibitors was slightly higher than the minimum dose required for NF-κB inhibition, indicating that NF-κB inhibition and apoptosis occur within the same dose range. Calpain I inhibitor was the most effective inhibitor of NF-κB, and PDTC was the least effective, reflecting the order of efficacy of these compounds in inducing apoptosis. Inhibition of NF-κB (maximal at 2 h) occurred prior to morphologic evidence of apoptosis (maximal at 18 h). This suggests that an event downstream of NF-κB inhibition (such as inhibition of gene transcription) would be required to provide a causal link between the two events.

### Discussion

We have shown that three pharmacologically distinct inhibitors of NF-κB – PDTC, TPCK and calpain I inhibitor – each causes apoptosis of RAW 264.7 murine macrophages in a dose-dependent fashion. Further, doses of these inhibitors which cause apoptosis are similar to those which inhibit NF-κB binding as detected by electrophoretic mobility shift assay. Simultaneous activation of the cells with either LPS or TNF fails to reverse apoptosis. Simultaneous treatment with PMA partially reverses apoptosis but the effect is only significant in calpain I inhibitor-treated cells.

The ability of TPCK, calpain I inhibitor and PDTC to cause apotosis has been previously documented in other cells lines,[14,34–37] but an association with inhibition of NF-κB had not been made until very recently when four simultaneous reports were published demonstrating a direct association between NF-κB inhibition and apoptosis.[24–27] Three reports (based on studies conducted in fibroblasts, macrophages, Jurkat T cells, human bladder cancer cells and human fibrosarcoma cells) showed that NF-κB activation suppresses TNF-α-induced apoptosis and that NF-κB inhibition (either by transfection with a dominant-negative I-κB alpha or by ‘knock-out’ of RelA), augments TNF-induced apoptosis.[24–26] Our study confirms these results in macrophages and extends them to unstimulated, and LPS-stimulated, cells. In a fourth study, inhibition of NF-κB by TPCK induced apoptosis in WEHI 121 B-cell lymphoma cells even in the absence of TNF-stimulation.[27] Interestingly, as in the case of RAW cells, WEHI cells have constitutive NF-κB activation.

TPCK and calpain I inhibitor are both protease inhibitors. TPCK is an inhibitor of serine chymotrypsin-like proteases and has been shown to inhibit both the phosphorylation and proteolytic degradation of I-κB.[38] Calpain I inhibitor is an inhibitor of neutral cysteine proteases and is known to prevent I-κB proteolytic degradation.[39] There is some evidence to suggest that calpain itself may be involved in the regulation of I-κB degradation.[40] However, it is also possible that the apoptosis-inducing effects of these two inhibitors could derive from their ability to inhibit the 26S proteasome, a multifraction protease which degrades and recycles a variety of intracelullar proteins. Indeed, the ability of PMA partially to reverse apoptosis induced by calpain I inhibitor may be related to its ability to activate the proteasome, most likely downstream from the effects of calpain I inhibitor. The proteasome's role in NF-κB activation is to degrade phosphorylated, ubiquitin-conjugated, cytoplasmic I-κB, therby releasing NF-κB dimers for nuclear binding.[41] The proteasome is also involved in the degradation of other proteins which are critical for cell cycle regulation including cyclins, p53 and protein kinase C.[42–44] Thus, it is not possible to determine from our data whether the effects of the two protease inhibitors tested here are due to the inhibition of NF-κB *per se*, or to inhibition of the degradation of another protein such as p53[36] involved in cell cycle regulation. However, in light of the recently published reports directly linking NF-κB inhibition to apoptosis by gene transfection,[24–26] it is likely that the effects of TPCK and calpain I inhibitor on apoptosis may be directly related to their ability to inhibit NF-κB transloction to the nucleus.

In some cell types, both TPCK and calpain I inhibitor have been shown to be effective inhibitors rather than inducers of apoptosis.[11,45–49] Their dual effects on apoptosis may derive from their ability to inhibit proteases involved in the characteristic nucleosomal cleavage of apoptosis independently of their ability to inhibit NF-κB.[45,46,49] Alternatively, it is possible that both underactivation and overactivation of NF-κB may each cause apoptosis, giving NF-κB inhibition a dual role in apoptosis. It is well known, for example, that both excessive cellular activation ('activation-induced' cell death) and insufficient cellular stimulation (growth-factor withdrawal) can each lead to cell death, most likely by different mechanisms. Finally, the divergent effects of these protease inhibitors on apoptosis may be dependent both on cell type and position in the cell cycle. The possibility exists, for example, that cycling cells are susceptible to apoptosis by these agents whereas terminally differentiated cells arrested in $G_0/G_1$ are not.

The mechanism by which PDTC inhibits NF-κB binding is still unclear. However, it has been suggested that its inhibitory effects of NF-κB are due to its metal-binding and/or antioxidant properties.[50,51] Oxidants may activate NF-κB through a mechanism independent of the proteasome. An oxidant-sensitive tyrosine kinase had recently been shown to release I-κB from NF-κB without associated I-κB degradation.[52] It is unlikely that PDTC's actions are similar to those of either TPCK or calpain I inhibitor. In unstimulated RAW cells, TPCK and calpain I inhibitor inhibited both bands of the NF-κB complex, presumably corresponding to p50–p65 and p50–p50 dimers. However, in our hands, PDTC only inhibited the higher molecular weight band corresponding to the p50–p65 dimer. In addition, administration of TPCK and calpain I inhibitor results in the appearance of a new unidentified band on EMSA of higher molecular weight than the p50–p65 heterodimer. The fact that PDTC only inhibits p50–p65 dimers but still induces apoptosis, supports the hypothesis, raised by findings in the p65 kock-out mouse,[21] that p65 but not p50 is critical for cell survival.

It is interesting that PDTC was substantially less effective than TPCK or calpain I inhibitor in inducing apoptosis in our cell line. One reason may be that higher doses of PDTC are required to inhibit NF-κB. In addition, anti-oxidants such as PDTC may be less potent inducers of apoptosis than protease inhibitors by virtue of their ability to scavenge free radicals. Finally, PDTC (but not, to our knowledge, TPCK or calpain I inhibitor) has the ability to induce metallothionein (unpublished work), a metal-regulatory protein which itself inhibits apoptosis.[53]

Our findings regarding PDTC's ability to induce apoptosis are consistent with the recently reported results of Tsai *et al.* who found that PDTC, at doses of 25–150 μM, induced apoptosis in rat and human aortic smooth muscle cells.[15] These authors concluded that PDTC's ability to induce apoptosis might be related to its antioxidant properties because similar results were obtained with *N*-acetyl cysteine. When serum-deprived PC12 cells were used in the same study, however, PDTC was protective at doses of 100 μM. It is clear from these divergent results that the actions of PDTC, like TPCK and calpain I inhibitor, are dependent on the cell type and cell cycle conditions. PDTC's ability to induce death in smooth muscle cells and macrophages, two cell types which proliferate on conditions of chronic inflammation, (while sparing nerve cells and possibly other terminally differentiated cells) suggests that PDTC and other NF-κB inhibitors might be useful anti-inflammatory agents.

Although the three compounds tested in this study inhibited NF-κB within 2 h, there was a time delay of 6 h before the appearance of apoptosis. This suggests that they are regulating gene transcription and protein translation of downstream effectors of cell survival or death. We used cycloheximide to test the hypothesis that the three compounds might be inducing the transcription of a programed cell death gene, since cycloheximide has been reported to block apoptosis caused by other agents.[33] Cycloheximide not only failed to block apoptosis caused by each of the three NF-κB inhibitors but instead exacerbated it. This suggests the alternative, and more likely, hypothesis that PDTC, TPCK and calpain I inhibitor are inhibiting the transcription of a gene required for cell survival. One candidate for the putative survival gene being repressed by the three inhibitors is A20, a zinc finger binding protein with an NF-κB site on the promoter known to protect cells from undergoing apoptosis.[24] Recent data from Beg and Baltimore, however, show that transfection of Rel A-deficient 3T3 cells with A20 is unable to prevent TNF-induced apoptosis.[24] In contrast, transfection of WEHI cells with c-*myc* and *Bcl-x<sub>L</sub>* can attenuate apoptosis induced by TPCK[27] suggesting that *Bcl-x<sub>L</sub>* and/or c-*myc*

may be downstream targets of NF-κB which promote cell survival.

In summary, our data from a transformed murine peritoneal macrophage cell line add to a recent and rapidly growing body of evidence linking NF-κB inhibition to induction of apoptosis in inflammatory and immune cells. Induction of apoptosis in immune effector cells may be a therapeutic mechanism of NF-κB inhibitors.

### References

1. Baeuerle P, Henkel T. Function and activation of NF-κB in the immune system. *Annu Rev Immunol* 1995; **12**: 141–179.
2. Sharma HW, Narayanan R. The NK-κB transcription factor in oncogenesis. *Anticancer Res* 1996; **16**: 589–596.
3. Higgins KA, Perez JR, Coleman TA, *et al.* Antisense inhibition of the p65 subunit of NF-κB blocks tumorigenicity and causes tumor regression. *Proc Natl Acad Sci USA* 1993; **90**: 9901–9905.
4. Franzoso G, Bours V, Park S, Tomita-Yamaguchi M, Kelly K, Siebenlist U. The candidate oncoprotein Bcl-3 is an antagonist of p50/NF-κB-mediated inhibition. *Nature* 1992; **359**: 339–342.
5. Ivanov VN, Deng G, Podack ER, Malek TR. Pleiotropic effects of Bcl-2 on transcription factors in T cells: potential role of NF-κB p50–p50 for the anti-apoptotic function of Bcl-2. *Int Immunol* 1995; **7**: 1709–1720.
6. Slater AF, Kimland M, Jiang SA, Orrenius S. Constitutive nuclear NF-κB/rel DNA-binding activity of rat thymocytes is increased by stimuli that promote apoptosis, but not inhibited by pyrrolidine dithiocarbamate. *Biochem J* 1995; **312**: 833–838.
7. Kuwakado K, Kubata M, Bessho R, *et al.* Augementation by aphidicolin of 1-beta-D-arbino-furanosylcytosine-induced c-jun and NF-κB activation in a human myeloid leukemia cell line: correlation with apoptosis. *Leuk Res* 1995; **19**: 645–650.
8. Tong L, Perez-Polo JR. Transcription factor DNA binding activity in PC12 cells undergoing apoptosis after glucose deprivation. *Neurosci Lett* 1995; **191**: 137–140.
9. Bessho K, Matsubara K, Kubata M *et al.* Pyrrolidine dithiocarbamate, a potent inhibitor of nuclear factor kappa B (NF-κB) activation, prevents apoptosis in human promyelocyte leukemia HL-60 cells and thymocytes. *Biochem Pharmacol* 1994; **48**: 1883–1889.
10. Lin KI, Lee SH, Narayanan R, Baraban JM, Hardwick JM, Ratan RR. Thiol agents and Bcl-2 identify an alphavirus-induced apoptotic pathway that requires activation of the transcription factor, NF-κB. *J Cell Biol* 1995; **131**: 1149–1161.
11. Higuchi M, Singh S, Chan H, Aggarwal BB. Protease inhibitors differentially regulate tumor necrosis factor-induced apoptosis, nuclear factor κB activation, cytotoxicity, and differentiation. *Blood* 1995; **86**: 2248–2256.
12. Park A, Baichwal VR. Systematic mutational analysis of the death domain of the tumor necrosis factor receptor 1-associated protein, TRADD. *J Biol Chem* 1996; **271**: 9858–9862.
13. Hsu H, Shu HB, Pan MG, Goeddel DV. TRADD–TRAF2 and TRADD–FADD interactions define two distinct TNF receptor 1 signal transduction pathways. *Cell* 1996; **84**: 299–308.
14. Hsu H, Huang J, Shu HB, Baechual V, Goeddel DV. TNF-dependent recruitment of the protein kinase RIP to the TNF receptor-1 signaling complex. *Immunity* 1996; **4**: 387–396.
15. Tsai JC, Jain M, Hsieh CM, *et al.* Induction of apoptosis by pyrrolidine dithiocarbamate and N-acetyl cysteine in vascular smooth muscle cells. *J Biol Chem* 1996; **271**: 3667–3670.
16. McCabe MJ, Orrenius S. Genistein induces apoptosis in immature thymocytes by inhibiting topoisomerase-II. *Biochem Biophys Res Commun* 1993; **194**: 944–950.
17. Trede NS, Castigli E, Geha RS, Chatila T. Microbiol superantigens induce NF-κB in the human monocytic cell line, THP-1. *J Immunol* 1993; **150**: 5604–5613.
18. Scheinman RI, Cogswell PC, Lofquist AK, Baldwin AS Jr. Role of transcriptional activation of I κB alpha in mediation of immunosuppression by glucocorticoids. *Science* 1995; **270**: 283–286.
19. Wyllie AH. Glucocorticoid-induced thymocyte apoptosis is associated with endogenous endonuclease activation. *Nature* 1980; **284**: 555–556.
20. Bertrand R, Solary E, O'Conner P. Kohn KW, Pommier Y. Induction of a common pathway of apoptosis by staurosporine. *Exp Cell Res* 1994; **211**: 314–321.
21. Ishikawa Y, Mukaida N, Kuno K, Rice N, Okamoto SI, Matsushima K. Establishment of lipopolysaccharide-dependent nuclear factor κB activation in a cell-free system. *J Biol Chem* 1995; **270**: 4158–4164.
22. Beg AA, Sha WC, Bronson RT, Ghosh S, Baltimore D. Embryonic lethality and liver degeneration in mice lacking the RelA component of NF-κB. *Nature* 1995; **376**: 167–170.
23. Sarma V, Lin Z, Clark L, *et al.* Activation of the B-cell receptor, CD40, induces A20, a novel zinc finger protein that inhibits apoptosis. *J Biol Chem* 1995; **270**: 12343–12346.
24. Beg AA, Baltimore D. An essential role for NF-κB in preventing TNF-α-induced cell death. *Science* 1996; **274**: 782–784.
25. Wang CY, Mayo MW, Baldwin AS Jr. TNF- and cancer therapy-induced apoptosis: potentiation by inhibition of NF-κB. *Science* 1996; **274**: 784–787.
26. Van Antwerp DJ, Martin SJ, Kafri T, Green DR, Verma IM. Suppression of TNF-α-induced apoptosis by NF-κB. *Science* 1996; **274**: 787–789.
27. Wu M, Arsura M, Bellas RE, *et al.* Inhibitors of c-myc expression induces apopotosis of WEHI 231 murine B cells. *Mol Cell Biol* 1996; **16**: 5015–5125.
28. Grilli M, Pizzi M, Memo M, Spano P. Neuroprotection by aspirin and sodium salicylate through blockade of NF-κB activation. *Science* 1996; **274**: 1383–1385.
29. Barnes PJ, Karin M. Mechanisms of disease: Nuclear factor-κB. A pivotal transcription factor in chronic inflammatory diseases. *New Engl J Med* 1997; **326**: 1066–1071.
30. Kruppa G, Thoma B, Machleidt T, Wiegmann K, Kronke M. Inhibition of tumor necrosis factor (TNF)-mediated NF-κB activation by selective blockade of the human 55-kDa TNF receptor. *J Immunol* 1992; **148**: 3152–3157.
31. Sgonc R, Boeck G, Dietrich H, Gruber J, Richeis H, Wick G. Simultaneous determination of cell surface antigens and apoptosis. *Trends Genet* 1994; **10**: 41–42.
32. Martin SJ. Apoptosis: suicide, execution or murder? *Trends Cell Biol* 1993; **3**: 141–144.
33. Martin DP, Schmidt RE, DiStefano PS, Lowry OH, Carter JG, Johnson EM. Inhibitors of protein synthesis and RNA synthesis prevent neuronal death caused by nerve growth factor deprivation. *J Cell Biol* 1988; **106**: 824–844.
34. Fearnhead HO, Rivett AJ, Dinsdale D, Cohen GM. A pre-existing protease is a common effector of thymocyte apoptosis mediated by diverse stimuli. *FEBS Lett* 1995; **357**: 242–246.
35. Sluvkin II, Jerrells TR. Different pathways of in vitro ethanol-induced apoptosis in thymocytes and splenic T and B lymphocytes. *Immunopharmacology* 1995; **31**: 43–57.
36. Shinohara K, Tomioka M, Nakano H, Tone S, Ho H, Kawashima S. Apoptosis induction resulting from proteasome inhibition. *Biochem J* 1996; **317**(2): 385–388.
37. Zhu W, Murtha PE, Young CY. Calpain inhibitor-induced apoptosis in human prostate adenocarcinoma cells. *Biochem Biophys Res Commun* 1995; **214**: 1130–1137.
38. Henkel T, Machleidt T, Alkalay I, Kronke M, Ben-Neriah Y, Baeuerle PA. Rapid proteolysis of IκB-α is necessary for activation of transcription factor, NF-κB. *Nature* 1993; **365**: 182–185.
39. Lin YC, Brown K, Siebenlist U. Activation of NF-κB requires proteolysis of the inhibitor IκB-α: signal-induced phosphorylation of IκB-α alone does not release active NF-κB. *Proc Natl Acad Sci USA* 1995; **92**: 552–556.
40. Liu ZQ, Kunimatsu M, Yang JP, Ozaki Y, Sasaki M, Okamoto T. Proteolytic processing of nuclear factor κB by calpain in vitro. *FEBS Lett* 1996; **385**: 109–113.
41. Chen Z, Halger J, Palombella VJ *et al.* Signal-induced site-specific phosphorylation targets I κB alpha to the ubiquitin-proteasome pathway. *Genes Dev* 1995; **9**: 1586–1597.
42. Friedman H, Snyder M. Mutations in PRGI, a yeast proteasome-related gene, cause defects in nuclear division and are suppressed by deletion of a mitotic cyclin gene. *Proc Natl Acad Sci USA* 1994; **91**: 2031–2035.
43. Maki CG, Huibregtse JM, Howley PM. In vivo ubiquitination and proteasome mediated degradation of p53(1). *Cancer Res* 1996; **56**: 2649–2654.
44. Lee HW, Smith L, Petit GR, Vinitsky A, Smith JB. Ubiquitination of protein kinase C-alpha and degradation by the proteasome. *J Biol Chem* 1996; **271**: 20973–20976.
45. Yoshida A, Takauji R, Inuzuka M, Ueda T, Nakamura T. Role of serine and ICE-like proteases in induction of apoptosis by etoposide in human leukemia HL-60 cells. *Leukemia Res* 1996; **10**: 821–824.
46. Mashima T, Naito M, Fujita N, Noguchi K, Tsuruo T. Identification of actin as a substrate of ICE and an ICE-like protease and involvement of an ICE-like protease but not ICE in VP-16-induced U937 apoptosis. *Biochem Biophys Res Commun* 1995; **217**: 1185–1192.
47. Mampuru LJ, Chen SJ, Kalenik JL, Bradley ME, Lee TC. Analysis of events associated with serum deprivation-induced apoptosis in C3H/So18 muscle satellite cells. *Exp Cell Res* 1996; **226**: 372–780.
48. Estaquier J, Tanaka M, Suda T, Nagata S, Golstein P, Ameisen JC. Fas-mediated apoptosis of CD4+ and CD8+ T cells from human immunodeficiency virus-infected persons: differential in vitro preventive effect of cytokines and protease antagonists. *Blood* 1996; **87**: 4959–4966.
49. Squier MK, Miller AC, Malkinson AM, Cohen JJ. Calpain activation in apoptosis. *J Cell Physiol* 1994; **159**: 229–237.

50. Meyer M, Schreck R, Baeuerle PA. $H_2O_2$ and antioxidants have opposite effects on activation of NF-κB and AP-1 in intact cells: AP-1 as secondary antioxidant-responsive factor. *EMBO J* 1993; **12**: 20005–20015.
51. Schreck R, Meier B, Mannel DN, Droge W, Baeuerle PA. Dithiocarbamates as potent inhibitors of nuclear factor κB activation in intact cells. *J Exp Med* 1992; **175**: 1181–1194.
52. Imbert V, Rupert RA, Livolsi A, *et al*. Tyrosine phosphorylation of IκB-α activates NF-κB without proteolytic degradation of IκB-α. *Cell* 1996; **86**: 787–798.
53. Sato M, Brenner I. Oxygen free radicals and metallothionein. *Free Rad Biol Med* 1993; **14**: 325–337.

ACKNOWLEDGEMENTS. This work was supported in part by NICHD 31885 awarded to M. J. S. Miller.

**Received 21 January 1997; accepted in revised form 14 May 1997**
